# Are MUC5B and TERT mutations genetic risk factors for pulmonary fibrosis in individuals with severe COVID-19?

**DOI:** 10.5578/tt.20239905

**Published:** 2023-03-10

**Authors:** N.A. Yetkin, A. Kiraz, B. Baran Ketencioğlu, C. Bol, N. Tutar

**Affiliations:** 1 Department of Pulmonogy, Erciyes University Faculty of Medicine, Kayseri, Türkiye; 2 Clinic of Medical Biology and Genetics, Kayseri Training and Research City Hospital, Kayseri, Türkiye; 3 Clinic of Pulmonogy, Kayseri Training and Research City Hospital, Kayseri, Türkiye

**Keywords:** Polymorphism, TERT, MUC5B, SARS-CoV-2

## Abstract

**ABSTRACT:**

Are MUC5B and TERT mutations genetic risk factors for pulmonary fibrosis in individuals with severe COVID-19?

**Introduction:**

The genetic risk factors for Coronavirus disease-2019 (COVID19)-associated pulmonary fibrosis (CAPF) are not clearly defined. Mutations in the genes encoding telomerase reverse transcriptase (TERT) and mucin 5B (MUC5B) are well-known genetic risk factors for pulmonary fibrosis. In this study, we aimed to show whether the most common proven mutations of pulmonary fibrosis affect the development of CAPF.

**Materials and Methods:**

Forty-eight patients who were matched for age, gender, COVID-19 disease severity, and respiratory support type and needed high flow nasal cannula, non-invasive mechanical ventilator, or invasive mechanical ventilator due to COVID-19 were followed up prospectively. Eighteen patients were excluded from the follow-up due to known structural lung disease, collagen tissue disease, and occupational exposure to fibrosis. The patients were called for follow-up three months after discharge, and CT was performed. Those with fibrosis (n= 15) in the third-month follow-up CT were included in the CAPF group, and those with complete resolution (n= 15) were included in the control group. Blood samples were taken for genetic analysis.

**Results:**

TERT gene study revealed that six (40%) of the fibrosis group was normal, while five were heterozygous (33.3%). MUC5B polymorphism was not detected in 10 (66.7%) of the fibrosis group.

**Conclusion:**

Individuals with TERT mutations may be at a higher risk for CAPF. Further studies are needed to clarify the genetic risk factors for CAPF.

## Abstract (tr)

**ÖZ:**

MUC5B ve TERT mutasyonları şiddetli COVID-19 olan bireylerde pulmoner fibrozis için genetik risk faktörleri midir?

**Giriş:**

Koronovirüs hastalığı-2019 (COVID-19) ile ilişkili pulmoner fibroz (CAPF) için genetik risk faktörleri net olarak tanımlanmamıştır. Telomeraz ters transkriptaz (TERT) ve müsin 5B (MUC5B) kodlayan genlerdeki mutasyonlar, pulmoner fibroz için iyi bilinen genetik risk faktörleridir. Bu çalışmada pulmoner fibrozisin kanıtlanmış en yaygın mutasyonlarının CAPF gelişimi üzerine etkisinin olup olmadığını göstermeyi amaçladık.

**Materyal ve Metod:**

Yaş, cinsiyet, COVID-19 hastalık şiddeti ve solunum destek tipi açısından uyumlu ve COVID-19 nedeniyle yüksek akımlı nazal kanül, non-invaziv mekanik ventilatör veya invaziv mekanik ventilatör ihtiyacı olan 48 hasta prospektif olarak izlendi. On sekiz hasta, bilinen yapısal akciğer hastalığı, kollajen doku hastalığı ve mesleki fibrozise maruz kalma nedeniyle takipten çıkarıldı. Hastalar taburcu olduktan üç ay sonra kontrole çağrılarak BT çekildi. Üçüncü ay kontrol BT’sinde fibrozis (n= 15) olanlar CAPF grubuna, rezolüsyonu tam olanlar (n= 15) kontrol grubuna eklendi. Genetik analiz için kan örnekleri alındı.

**Bulgular:**

Fibrozis grubunun altısında (%40) TERT gen analizi normal, beşinde (%33,3) heterozigot bulundu. Fibrozis grubunun 10’unda (%66,7) MUC5B polimorfizmi saptanmadı.

**Sonuç:**

TERT mutasyonları olan kişiler CAPF için daha yüksek risk altında olabilir. CAPF için genetik risk faktörlerini netleştirmek için daha fazla çalışmaya ihtiyaç vardır

## Introduction

In December 2019, reports of severe acute respiratory disease caused by severe acute respiratory syndrome coronavirus-2 (SARS-CoV-2) began to emerge from Wuhan, China. As of January 2022, more than 300 million people had been infected with SARS-CoV-2 and more than five million people died due to Coronavirus disease-2019 (COVID-19) ( 1 ). However, the long-term multisystem morbidities associated with COVID-19 have not been fully documented, and concerns remain, especially regarding long-term pulmonary complications. It has been shown that more than one-third of patients recovering from COVID-19 developed fibrotic abnormalities at discharge. In a meta-analysis examining postCOVID-19 long-term effects, the development of pulmonary fibrosis was reported as 5% ( 2 ). In a very recently published meta-analysis, COVID-19- associated pulmonary fibrosis (CAPF) development was reported as 44.9% ( 3 ).Pulmonary fibrosis (PF) can develop either following chronic inflammation or as a primary, genetic, and age-related fibroproliferative process, as in idiopathic pulmonary fibrosis (IPF). Post-viral fibrosis and physiological deterioration are known consequences of previous coronavirus outbreaks; therefore, it is predictable that the global burden of fibrotic lung disease may increase in the coming years after the COVID-19 pandemic. Radiological findings are variable throughout the condition, and persistent computed tomographic (CT) imaging abnormalities up to day 37 have been reported in the literature ( 3 ). Early diagnosis of patients at high risk for CAPF is critical for determining treatment strategies. Recent evidence suggests that the predictors of CAPF are similar to IPF ( 4 ). Ojo et al. concluded that the predictors of CAPF include advanced age, illness severity, length of intensive care unit stay and mechanical ventilation, smoking, and chronic alcoholism ( 5 ).

Additionally, leukocytosis, neutrophilia, lymphopenia, eosinopenia, elevated CRP, and D-dimer were defined as laboratory predictors for CAPF ( 6 ). However, genetic markers that pose a risk for fibrosis are not well defined. Mutations in the genes encoding telomerase reverse transcriptase (TERT) ( 7 ) and mucin 5B (MUC5B) are well-known risk factors for pulmonary fibrosis.

To the best of our knowledge, well-known genetic risk factors for pulmonary fibrosis, TERT, and MUC5B mutations, have not been evaluated in individuals with CAPF. The present study aims to explore the role of mutations of TERT and MUC5B genes in the development of CAPF.

## MATERIALS and METHODS

### Participants

This study was conducted in accordance with the Helsinki Declaration. Ethics Committee approval was obtained from Kayseri City Training and Research Hospital (Ethics Committee Meeting Date: 08.06.2020, Decision No: 76397871/39) and the study was funded by Kayseri City Training and Research Hospital. Patients in two tertiary centers, Kayseri City Training and Research Hospital and Erciyes University Faculty of Medicine, were classified as severe and critical COVID-19, either with a positive swab PCR test or a negative swab PCR test, with clinical and thoracic CT findings compatible with COVID-19. Forty-eight patients who were matched for age, gender, COVID19 disease severity, respiratory support type, and COVID-19 treatment and who needed high flow nasal cannula, non-invasive mechanical ventilator, or invasive mechanical ventilator due to COVID-19 were followed up prospectively. Eighteen individuals with connective tissue disease, a history of silica exposure, radiotherapy, and drug use known to cause pulmonary fibrosis were excluded from the study. Follow-up CT was performed three months after discharge (n= 30). Individuals (n= 15) with diffuse and permanent fibrotic changes such as parenchymal bands, irregular interfaces, reticular opacities, traction bronchiectasis, and honeycomb appearance on follow-up CT, as previously described ( 6 , 8). ), were included in the CAPF group; those whose CT recovered completely were taken as the control group; and had no fibrosis findings in lung CT scan in the third month after discharge were included in the study as the control group. Blood biomarkers for connective tissue diseases were negative in the patient and control groups. Also, blood samples were taken for TERT and MUC5B genetic analysis.

### Genetic Analysis

In this study, severe COVID-19 patients were followed prospectively. Peripheral blood samples were taken after consent was obtained from the patients. Genomic DNA was extracted from peripheral blood samples using the DNA isolation kit according to the manufacturer’s instructions (DETAGEN Whole Blood DNA Isolation Kit, DETAGEN Genetic Corp, Türkiye). Density (ng/µL) and absorbance measurements (A260/280; 1.80-2.00) of DNA samples were performed with Nanodrop Lite (Thermo Scientific, USA). MUC5B (rs35705950) and TERT (rs2736100) SNP were investigated. The real-time PCR study was performed using the DETAGEN MUC5B real-time PCR kit and the DETAGEN TERT real-time PCR kit (DETAGEN Genetic Corp, Türkiye). In line with kit protocols: For MUC5B, 2 µL (20-100 ng) of genomic DNA, mix-1 (6 µL), mix-2 (1 µL), primer probe mix (2.5 µL) and nuclease-free water (1 µL), total volume 12.5 µL, were taken into optical capped PCR tubes and prepared for real-time PCR. For TERT, 2 µL (20- 100 ng) of genomic DNA, mix-1 (6 µL), mix-2 (1 µL), primer probe mix (2.5 µL), and nuclease-free water (1 µL), total volume 12.5 µL, were taken into optical capped PCR tubes and prepared for real-time PCR. Real-time PCR conditions were set as 40 cycles of 95℃ 10 minutes, 95℃ 10 seconds, 56℃ 10 seconds, and 72℃ 20 seconds (fluorescent reading). According to the kit procedure, the work was performed with the CFX96 Touch real-time PCR detection system (Bio-Rad CFX, USA). After the real-time PCR process was completed, the analysis was evaluated in Bio-Rad CFX Manager software (Bio-Rad CFX, USA). For analysis, two-channel reading was performed according to the kit protocol, and the FAM channel was evaluated as “wild type” and the HEX channel as “mutant.”

### COVID-19 Treatment

Intensive care specialists administered favipiravir, steroid, and tocilizumab combinations for the treatment of COVID-19. Favipiravir was given as a loading dose of 1800 mg BID on day one, followed by 800 mg BID from day two to fourteen. Steroid treatment was administered intravenously for ten days and then orally by dose tapering. Initially, tocilizumab was administrated at 8 mg/kg (up to 800 mg per dose) intravenously and the dose was repeated after 12 hours if needed.

### RESULTS

The median age was 63 (50-64) in the CAPF group and 56 (51-63) in the control group (p= 0.633). There was no significant difference between CAPF and control groups regarding age, gender, smoking status, and BMI (p= 0.633, 0.690, 0.395, and 0.687, respectively). In the CAPF group, three patients received high-flow oxygen, six received non-invasive mechanical ventilation support, and six were mechanically ventilated. In the control group, four patients received high-flow oxygen, five received non-invasive mechanical ventilation support, and six were mechanically ventilated. There were no statistical differences between groups in terms of respiratory support (p= 0.890). All individuals received favipiravir for COVID-19 treatment. Three patients in the CAPF group and two patients in the control group received favipiravir, steroids, and tocilizumab. Six patients in the CAPF group and seven patients in the control group received favipiravir and steroids. Six patients in each group received favipiravir alone. COVID-19 treatment protocols were similar in each group (p= 0.871). The demographic and clinical characteristics of individuals are presented in Table ( Table 1 ).

**Table 1:** Demographic and clinical characteristics of individuals with or without CAPF

Age (year)	63 (50-64)	56 (51-63)	0.633*
Gender, n (%)			
Female	5 (33.3)	25 (26.7)	0.690#
Male	10 (66.7)	20 (73.3)	
BMI	27.7 ± 4.2	26.3 ± 3.7	0.395*
Smoking status, n (%)			
Nonsmoker	7 (46.7)	9 (60)	0.687#
Smoker	3 (20.0)	3 (20)	
Ex-smoker	5 (33.3)	3 (20)	
Respiratory support type, n (%)			
High Flow Oxygen	3 (20)	4 (26.7)	
NIMV	6 (40)	5 (33.3)	0.890#
Mechanical ventilation	6 (40)	6 (40)	
COVID-19 treatment			
FAV alone	6 (40)	6 (40)	
FAV + Steroids	6 (40)	7 (46.7)	0.871#
FAV + Steroids + TCZ	3 (20)	2 (13.3)	

Values are presented as mean ± standard deviation, median (IQR, 25th-75th percentile), and n (%). *Mann-Whitney U test, #Pearson Chi-square test, BMI: Body mass index, NIMV: Non-invasive mechanical ventilation, FAV: Favipiravir, TCZ: Tocilizumab.

Telomerase reverse transcriptase gene analysis was normal in six (40%) of the CAPF group, while heterozygous mutation was found in five (33.3%), and homozygous mutation was found in four (26.7%). In the control group, seven (46.7%) did not have TERT gene polymorphism, while seven (46.7%) were heterozygous, and one (6.7%) had homozygous polymorphism. While MUC5B polymorphism was not detected in 10 (66.7%) patients in the CAPF group, heterozygous mutations were detected in five (33.3%). MUC5B homozygous polymorphism was not detected in any individual in the CAPF group. In the control group, 10 (66.7%) had no polymorphism in the MUC5B gene, while four (26.7%) had heterozygous and one (6.7%) had homozygous polymorphism. There was no statistically significant

**Figure 1:**
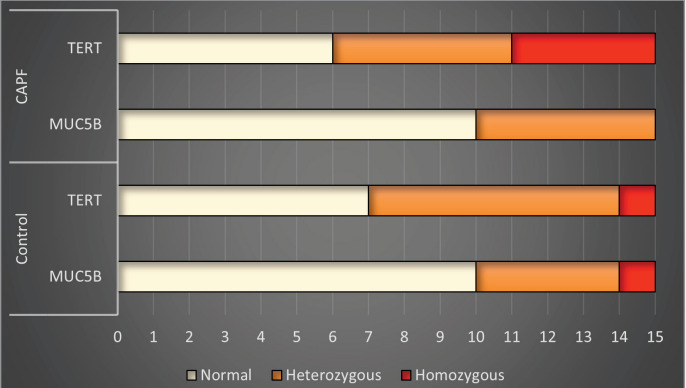
Analysis of MUC5B and TERT genes among study groups.

**Table 2:** Genetic characteristics of individuals with or without CAPF

TERT genetics, n (%)			
Normal	6 (40.0)	7 (46.7)	
Heterozygous	5 (33.3)	7 (46.7)	0.331#
Homozygous	4 (26.7)	1 (6.7)	
MUC5B genetics			
Normal	10 (66.7)	10 (66.7)	
Heterozygous	5 (33.3)	4 (26.7)	0.574#
Homozygous	0 (0.0)	1 (6.7)	

Values are presented as mean ± standard deviation, median (IQR, 25th-75th percentile), and n (%). *Mann-Whitney U test, #Pearson Chi-square test.

difference between the fibrosis and control groups in terms of TERT and MUC5B polymorphisms (p= 0.331 and p= 0.574) (Figure 1 and (Table 2).

### DISCUSSION

For the first time, two well-known genetic risk factors for pulmonary fibrosis were evaluated for the development of CAPF in this study. Two patient groups with similar COVID-19 disease severity and treatment modalities were compared in terms of TERT and MUC5B genetic analysis. We showed that TERT genetic abnormalities were slightly higher in the CAPF group, while MUC5B genetic analyses were similar between groups.

Findings on the long-term sequelae of COVID-19 have only recently begun to be explored, but there has been little research on post-COVID-19 lung sequelae to date. Here we describe the characteristics of the patients admitted to the intensive care unit with severe or critical COVID-19 and then recovered, with residual lung disease three months after discharge. We also investigated the relationship between TERT and MUC5B mutations and CAPF development.

The aim was to elucidate the cause and type of genetic predisposition that may lead to the development of pulmonary fibrosis with permanent functional deficit observed in long-term follow-up, as in H1N1 and MERS-CoV pneumonia ( 9 , 10 ).

In our study, the frequency of CAPF was observed to be higher in elderly patients, similar to the literature ( 3 ). Unlike the literature, we did not include cases that could cause pulmonary fibrosis such as chronic obstructive pulmonary disease, occupational exposure, and collagen tissue disease. Since we included severe COVID-19 patients with intensive care hospitalization in our study, we predicted that resolution might be delayed in severe pneumonia and took the 3rd month after discharge as the cut-off point for follow-up CT scans. In another study in which steroid treatment was given after COVID-19, six-week controls after discharge were taken as the basis. Patients with a milder disease did not require more than 24 hours of oxygen therapy and did not require intensive treatment care ( 11 ). As a result, as we assumed that the resolution time would be delayed due to the severity of the disease, we took the third month following discharge as the starting point.

Myall et al. found a radiological appearance compatible with organizing pneumonia at a rate of 59% in the post-COVID-19 period ( 11 ). In our study, no distinction was made for the dominance of interstitial appearances.

Previously, post-COVID-19 patients were treated with steroids, and a significant improvement was observed in their functional parameters within three weeks ( 11 , 12 ). Since we examined the patients genetically in our study, we followed up only with imaging; we did not subject them to functional and spirometric examination. In addition, it has been reported on a case-by-case basis that steroid treatment was given to a patient who developed post-COVID-19 fibrosis ( 12 ).

Early identification of patients at high risk for CAPF is crucial for determining treatment strategies. Recent evidence suggests that predictors of CAPF have similar pathogenesis to pulmonary fibrosis. In this study, the effect of MUC5B and TERT genes on CAPF was investigated for the first time, and the contribution of inflammatory markers to the process was investigated.

Consistent with the literature, the fibrosis group was older, disproportionately male, and had more comorbidities ( 11 ). This supports the association of advanced age with TERT mutation.

As shown in Figure 1, the frequency of TERT gene polymorphism increased in the fibrosis group, even though it was not statistically significant in our study. The small number of patients might be a limitation that caused the TERT gene polymorphism to be statistically insignificant. However, MUC5B gene polymorphism, unlike IPF, appears to be similar between the groups in our study.

The limitation of the present study is that it is not clear when exactly imaging should improve in postCOVID-19 patients. There is no clear data on this in the literature. According to our observations, complete resolution can be observed in the third month, even in severe and critical COVID-19 patients. This will necessitate more in-depth research. The results were not statistically significant due to the small number of patients.

Many factors affect the development of pulmonary fibrosis in individuals with COVID-19. Pulmonary fibrosis associated with viral infections can be triggered by age, direct cellular damage, increased cytokine levels, induction of the profibrotic pathway by viral antigens, and mechanical ventilation trauma. In addition, high oxygen supplement is an important cause of CAPF ( 13 ). In this study, clinical differences between the patient and control groups were tried to be minimized. As a limitation of the study, the duration of oxygen support and the percentage of F_i_O^2^ given to the patients in the CAPF and control groups could not be documented. However, the fact that the patients in both groups had similar COVID19 severity and received similar types of respiratory support suggests that this difference can be ignored. It is also unknown whether there are differences in cytokine levels between the groups. Similarly, it can be assumed that there are no significant differences in cytokine levels between groups whose COVID-19 disease severity and treatments were quite similar.

Despite the limitations of our study, there is a relatively large number of patients with CAPF, which is a rare condition. Also, this study provides important information in the identification of individuals who are genetically predisposed to CAPF.

### CONCLUSION

Individuals with TERT mutations, in particular, may be at a higher risk of developing CAPF. More research is needed to determine the genetic risk factors for CAPF.

### Ethical Committee Approval:

Ethics Committee approval was obtained from Kayseri City Training and Research Hospital (Ethics Committee Meeting Date: 08.06.2020, Decision No: 76397871/39).

### Conflict of INTEREST

The authors declare that they have no conflict of interest.

### AUTHORSHIP CONTRIBUTIONS

Concept/Design: NAY, NT, BBK, AK

Analysis/Interpretation: NAY, CB, NT

Data acqusition: NAY, BBK, CB, NT

Writing: NAY, AK, BBK, NT

Clinical Revision: NAY, NT, AK

Final Approval: NAY, NT, AK, BBK
